# Spatially heterogenous dynamics in colloidal gels during syneresis†

**DOI:** 10.1039/d3sm00448a

**Published:** 2023-06-29

**Authors:** Qimeng Wu, Jesse Buijs, Sanne de Groot, Hanne M. van der Kooij, Jasper van der Gucht, Thomas E. Kodger

**Affiliations:** a Physical Chemistry and Soft Matter, Wageningen University and Research, Stippeneng 4 6708 WE Wageningen The Netherlands thomas.kodger@wur.nl

## Abstract

Syneresis, the compaction of a material accompanied by fluid expulsion, is a typical mechanical instability which exists among colloidal gel based materials and that negatively affects the quality of relevant applications. We shed light onto the internal dynamics of model colloidal gels undergoing syneresis using Laser Speckle Imaging (LSI). The resulting dynamical maps capture the distinct differences in spatial and temporal relaxation patterns between colloidal gels comprising solid and liquid particles. This indicates different mechanisms of syneresis between the two systems and highlights the importance of the constituent particles and their mobile or restrictive interfaces in the mechanical relaxation of the colloidal gels during syneresis.

## Abstract 2

Colloidal gels composed of viscous particles undergoing syneresis exhibit heterogeneous mobility as visualized by laser speckle imaging.

## Graphical abstract

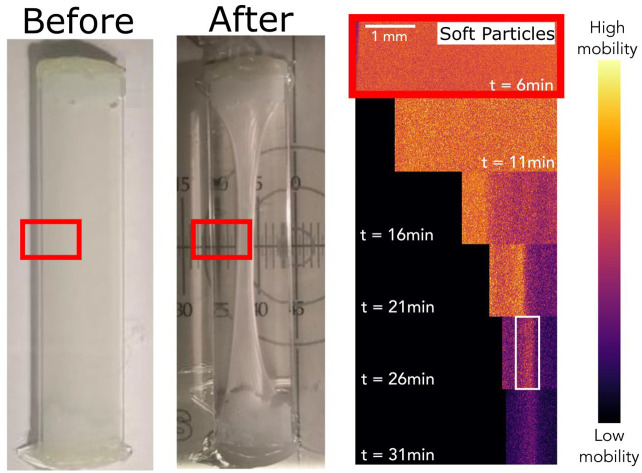

## 1 Introduction

Colloidal gels are soft solids in which attractions between colloidal particles drive a thermodynamic instability that through aggregation results in a space-spanning network structure of which the mechanical and transport properties can be suitably tuned.^1,2^ Colloidal gels are widely encountered in applications such as drug delivery systems, food products, coatings, and more.^3,4^ While a colloidal gel forms, particles tend to maximize contact with each other to minimize the interfacial energy cost in the network; this leads to local, limited compaction in the structure resisted by the network elasticity. The heterogeneity of this compaction leads to randomly distributed regions of inhomogeneous deformations, each of which generates a displacement field around it due to the elasticity of the network. These inhomogeneities can be considered as localized internal stress built within the network.^5,6^ When this internal stress is of sufficient amplitude or is facilitated by external stress, colloidal gels may suffer from a specific mechanical failure known as syneresis,^7,8^*i.e.* the contraction of the material accompanied by the expulsion of fluid. Syneresis is a typical mechanical instability encountered in food colloids, which is characterized by the appearance of fluid after a scoop in products like yoghurt and low-fat manufactured food. Unlike yielding of a solid network and fluidization of a gel induced by applied strains, the syneresing material itself does not necessarily undergo a solid to liquid transition, which indicates that the governing mechanisms are different than strain induced yielding.

Syneresis has been studied most intensively with macroscopic samples such as cheese and yoghurt, a colloidal gel formed by casein micelles. To date, the investigations have been centered on bulk syneretic properties, *i.e.* dimensional changes, rate of fluid expulsion and structural rearrangement obtained indirectly by rheology.^9^ Correlations have been found between the expulsion of fluid and parameters like pH and temperature.^7,10,11^ Additionally, internal and external stresses applied to the material robustly influence the magnitude of syneresis.^12,13^ Rearrangements within the network play a crucial role in determining the ease of fluid expulsion; such structural changes have been probed using rheology, with tan *δ* representing the ratio of dissipated energy due to interparticle bond breaking and stored elastic energy caused by bond extension.^10,14^ Such ensemble measurements, however, do not capture the spatial heterogeneity of a material undergoing syneresis. More specifically, syneresis is not purely a surface phenomenon, rather, it occurs over an unknown distance into the sample and over an unknown time scale. When syneresis occurs, while the material may retain its initial shape and does not appear to flow on the macroscopic scale, on the microscopic scale, the material must undergo rearrangements leading to progressive fluid expulsion. Elucidating a deeper causation between microscopic dynamics and bulk syneresis in a colloidal gel is a crucial step towards understanding the mechanical response of such colloidal materials. However, determining how the fluid and particle dynamics at the microscopic level define syneresis is challenging due to the wide range of time and length scales involved. Additionally, conventional optical microscopy is often inadequate for this task as particle dispersions such as colloidal gels and similar commercial products cause multiple scattering of light and loss of light transmission.

Therefore in this work, we apply a light scattering technique, Laser Speckle Imaging (LSI), which utilizes the turbidity to resolve the dynamics within non-transparent materials and is the imaging counterpart of Diffusive Wave Spectroscopy.^15,16^ LSI was initially developed to visualize the subcutaneous flow of blood in medical imaging in 1980s and has recently gained popularity in exploring dynamic phenomena like self-healing of polymers and delayed fracture in soft solids.^17–22^ LSI relies on coherent illumination of an optically turbid material. Backscattered light, coming from multiple scatter events of photons, is then detected by a camera. The interference of these scattered waves creates a two dimensional pattern known as a speckle image. In an evolving sample, such as a colloidal gel undergoing syneresis, the dynamics of the speckle pattern change which creates contrast for analysis.^23,24^ LSI thus uses changes in photon path-length resulting from nanoscale particle movement at millisecond to hour time scales and micron to centimeter spatial dimensions. We use LSI to create highly resolved dynamic maps of model colloidal gels during syneresis. These measurements allow for identification and characterization of the microscopic dynamics that govern syneresis. We find that the spatial dynamics evolve as the dispersion undergoes syneresis with some syneresing samples developing a region of high mobility. Interestingly, the presence of this region depends on the nature of the colloidal particle comprising the colloidal gel; viscous particles, similar to oil droplets in food products, exhibiting this region while solid particle do not.

## 2 Results and discussion

### 2.1 Spatiotemporal analysis of syneresis *via d*_2_

Colloidal dispersions consisting of polystyrene particles (PS) or polybutylacrylate particles (PBA) with an averaged radius, 〈*r*〉 = 275 nm, at *ϕ* = 0.01 and 0.02 respectively, in aqueous density matched solution are prepared in non-adhesive capillaries; volume fractions are chosen such that the elastic shear modulus, *G*′, after gelation is identical for both dispersions.^25^ Both types of particles have 0.1 wt% of a thermoresponsive surfactant adsorbed onto their surface. Upon heating to 32 °C, reversible and temperature-triggered adhesive forces are induced between the particles, with an attractive strength ≫*k*_B_*T*,^26,27^ leading to rapid colloidal gelation. Immediately after formation, gels undergo compaction, expelling the continuous fluid as seen in the recorded Movies S1 and S2 (ESI).

**Fig. 1:**
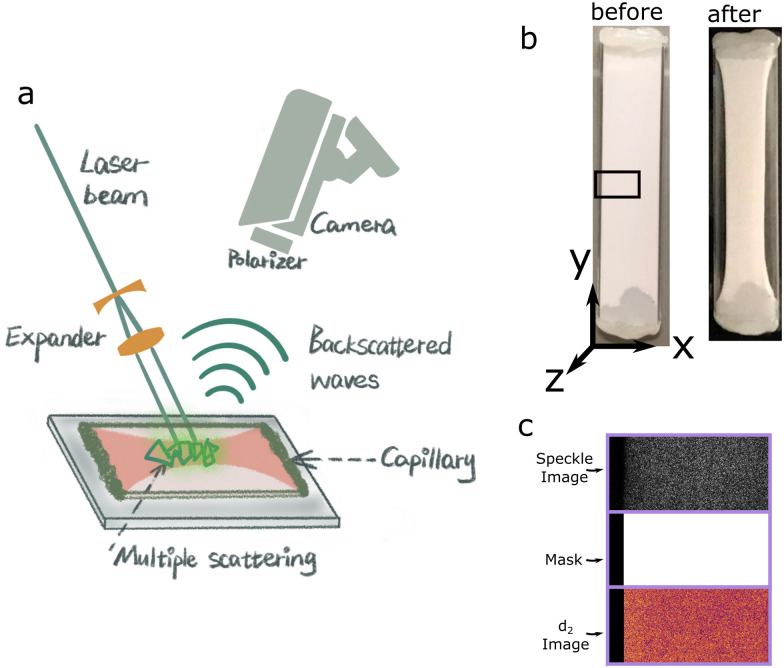
(**a**) Schematic illustration of back-scatter LSI set-up. The syneresing sample is illuminated with a coherent plane light wave, which is multiply scattered by the turbid sample, giving rise to a raw speckle image captured by the camera, modified from;^21^ (**b**) capillary containing PS sample before and after syneresis – capillary orientation [*x*,*y*,*z*] is indicated with dimensions [9,40,0.9] mm; syneresis occurs in the *x*-direction. The solid black box represents the LSI field of view; (**c**) Example images of a raw speckle image, a binary threshold mask and resultant image of multiplying the mask with the *d*_2_ map. Each purple rectangle represents one image.

We start an LSI measurement by heating a climate chamber which contains all of the optical elements.^16^ After the temperature in the climate chamber reaches 32 °C, the sample is inserted and data collection begins. A coherent light illuminates the sample and back scattered light is captured by a camera, illustrated in Fig. 1(a). The orientation of the LSI field of view respective of the capillary is shown in Fig. 1(b) with the dispersion syneresing in *x* direction due to the adhesive boundary conditions at the openings of the capillary in the *y* direction. Photon path length differences within the turbid sample generate a speckle pattern as shown in Fig. 1(c). While the absolute intensities in the snapshots of the speckle pattern are irrelevant, information on the internal dynamics of the colloidal gel during syneresis in time and space is extracted from analyzing the temporal fluctuations of the raw speckle images *via* the contrast function *d*_2_ (*t*, *x*, *y*, *τ*)^16,28,29^1
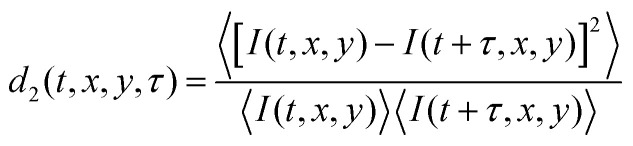
with 〈…〉 indicating averaging over time, where *I* is the speckle intensity at position (*x*, *y*), *t* is the age of the sample which provides the temporal dimension and *τ* sets the time lag between which images are autocorrelated, and hence the relaxation time of the dynamics of focus; small values of *τ* correspond to fast, high-frequency dynamics, while large values of *τ* enable the study of low-frequency processes. Based on *d*_2_, spatiotemporal maps of the dynamics can be created.^30,31^ High *d*_2_ values indicate decorrelation of the signal and hence signify enhanced local mobility. Fluctuations occur in the void area in the field of view, thus the *d*_2_ maps are multiplied with a binary threshold mask, shown in Fig. 1(c), to eliminate these fluctuations.

**Fig. 2:**
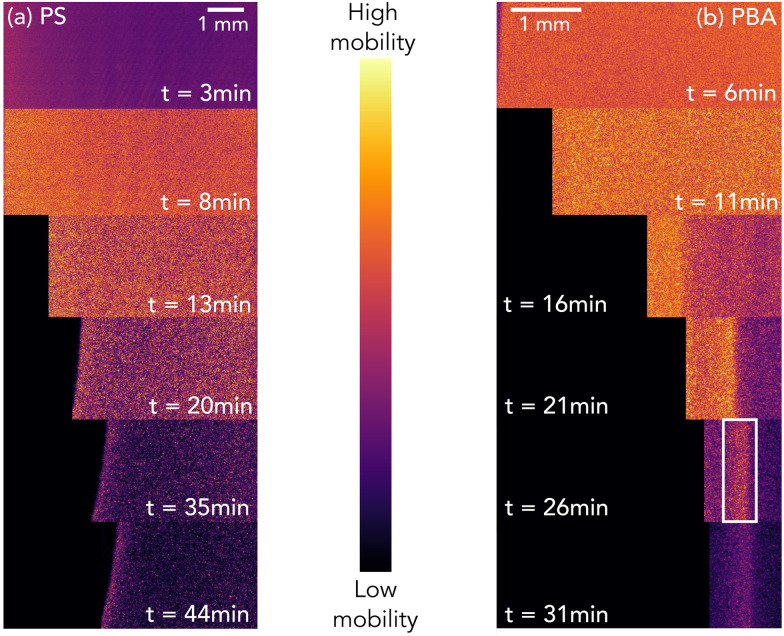
Time-lapse *d*_2_ maps of (**a**) the PS and (**b**) the PBA gel during syneresis, *τ* = 0.125 s. Left end of *d*_2_ maps represents the capillary wall. Note the activity band in the PBA gel indicated with a white rectangle for *t* = 26 min. Note: the *x*-dimension of (b) has been reduced to better show the activity band as shown in Fig. 1b.

We monitor the syneresis process over time with LSI and compute *d*_2_ values which encode the dynamics of the colloidal gel during syneresis, as shown in Fig. 2. These *d*_2_ maps of PS and PBA colloidal gels for *τ* = 0.125 s are shown as an example of the temporal development of dynamics during syneresis, where *t* = 0 min is defined as the moment when *T* = 32 °C. The edge of the colloidal gel corresponds to the capillary wall at *t* = 0 min and moves as syneresis occurs. Compared with the PS gel, a more substantial syneresis is observed in the PBA gel, which manifests in higher *d*_2_ values. During the first 8 minutes for the PS gel (Fig. 2(a)), mobility increases within the entire sample while there is no sign yet of macroscopic syneresis as the gel is still forming. When syneresis proceeds, the colloidal gel detaches from the capillary wall and mobility in the bulk decreases, while around the gel edge a relatively high mobility remains. For the PBA gel (Fig. 2(b)), higher mobility emerges after 6 minutes, indicated by the higher *d*_2_ value. The mobility near the moving gel edge remains high while the mobility in the bulk starts to decrease after 16 minutes, which is similar to the PS gel but this higher dynamic activity region extends over a wider range and occurs much earlier. Meanwhile, a zone of increased dynamic activity develops behind the gel edge, which lingers until the end of syneresis (Fig. 2(b), white rectangle). After 26 minutes, the rate of syneresis slows down and *d*_2_ at the gel edge decreases as well. In general, the mobility in the bulk slows down faster than the mobility near the edge, suggesting that there is spatial dependence of the dynamics during relaxation. Dissimilar to previous reports,^32^ no subsequent swelling is seen in either syneresing dispersions, and thus the gel surface remains topologically flat and smooth with no wrinkling or buckling.

### 2.2 Spatial relaxation with different relaxation times

**Fig. 3:**
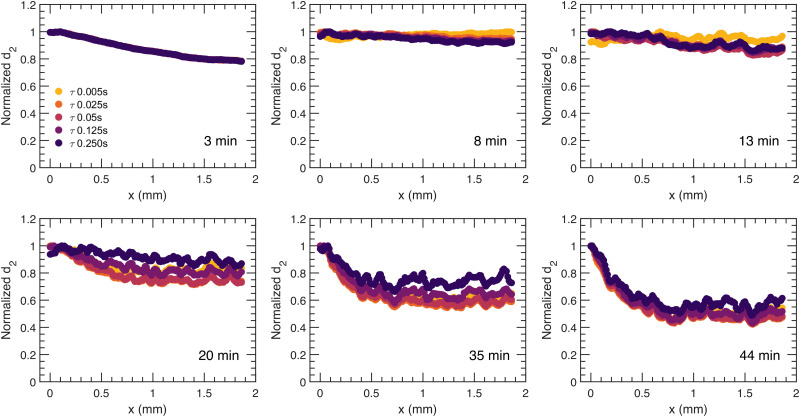
Profiles of normalized *d*_2_ for the PS colloidal gel at relaxation times, *τ* = 0.005, 0.025, 0.05, 0.125, 0.250 s as a function of distance to the moving gel edge for increasing sample age, *t* = 3, 8, 13, 20, 35, 44 minutes. *x*(*t*) = 0 mm represents the edge of the gel at the corresponding age. To highlight the spatial dependence, a normalized *d*_2_ value is used as the absolute *d*_2_ changes dramatically as the gel forms, synereses, and subsides.

**Fig. 4:**
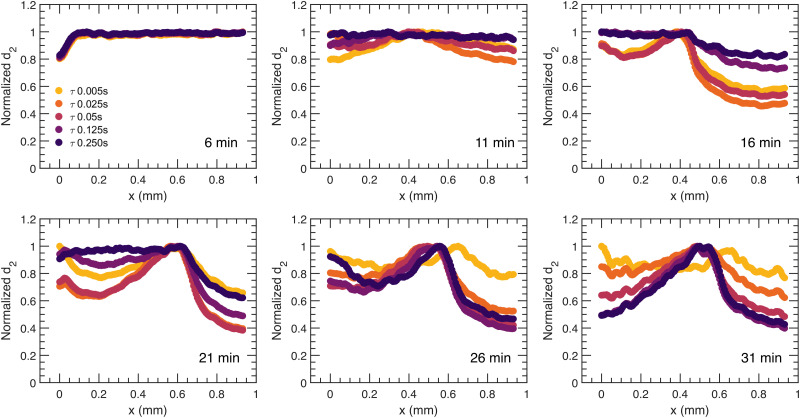
Profiles of normalized *d*_2_ for the PBA colloidal gel at relaxation times, *τ* = 0.005, 0.025, 0.05, 0.125, 0.250 s as a function of distance to the moving gel edge for increasing sample age, *t* = 6, 11, 16, 21, 26, 31 minutes. *x*(*t*) = 0 mm represents the edge of the sample at the corresponding age. To highlight the spatial dependence, a normalized *d*_2_ value is used as the absolute *d*_2_ changes dramatically as the gel forms, synereses, and subsides.

For a more quantitative description of the spatial dependence of the local dynamics in the direction of syneresis as observed in Fig. 2, a normalized *d*_2_, with the value of *d*_2_ being divided by the relative maximum for that sample and averaged along the *y* dimension, is computed and displayed as a function of distance to the moving gel edge, *x*, for different times after gelation, *t*, and for different correlation times, *τ*, in Fig. 3 and 4, for PS and PBA gels, respectively. Importantly, this *d*_2_ analysis assumes that the moving edge of the sample is slow relative to the maximum correlation time *τ*; indeed, for both PS and PBA samples, with maximum edge translation velocities of 3 μm s^−1^, this translation corresponds to less than one camera pixel and thus the speckle mobility, *i.e. d*_2_, is not a result of the edge translation. As shown in Fig. 3, for the PS gel, the dynamic activity decreases gradually away from the edge in a similar manner for all *τ*, implying that dynamic processes have similar spatial dependence. In contrast to the dynamics in the PS gel, the normalized spatial *d*_2_ profile is much more diverse in the PBA gel (Fig. 4), showing a band of enhanced dynamic activity a few hundred microns behind the edge of the gel, which we call the activity band (Fig. 2(b), white rectangle). The activity band already rises for small *τ* at 11 minutes and appears in all *τ* after 21 minutes. Additionally, the spatial *d*_2_ pattern shows a dependence on *τ*, which suggests that in several locations different dynamic processes occur such as diffusion and ballistic or advective motion as a result of the compaction. We will investigate those differences through a mean squared displacement analysis in Section 2.3.

**Fig. 5:**
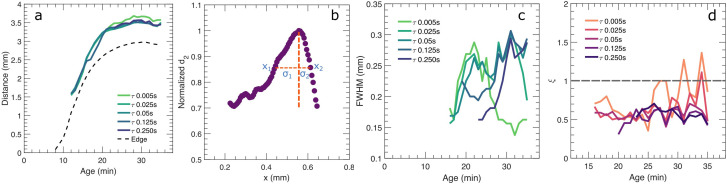
(a) Distance from the moving gel edge of the PBA gel (dashed line) and peak of the activity band (solid lines) to the glass capillary wall: the shrinkage of the sample over time is displayed together with the location of the peak of the activity band, as a function of age, *t*. (**b**) Example for determination of FWHM and symmetry ratio. The FWHM is the length between points annotated as *x*_1_ and *x*_2_ occurring at half of the range of the normalized *d*_2_, *i.e. d*_2_ = 0.85. (**c**) FWHM of the activity band over different *τ* as a function of *t*. (**d**) The asymmetry of the activity band distribution, represented by *ξ* = *σ*_2_/*σ*_1_, at different *τ* as a function of *t*.

To investigate the distinct spatial dependence of *d*_2_ between the two types of colloidal gels, we take a closer look at the activity band in the PBA gel. Firstly, the position of the peak of the band and the moving gel edge with respect to the capillary wall is calculated; the macroscopic motion of the whole gel is substantial, as indicated by the distances from the moving gel edge to the capillary wall as a function of *t* in Fig. 5(a) (dashed line). For all calculated *τ*, the activity band grows further away with respect to the moving gel edge in the first 20 minutes and retains the same distance to the gel edge as the macroscopic movement of the gel subsides which is indicated by the plateau for the later ages in Fig. 5(a) (dash line).

The shape of the activity band also changes over time as seen in Fig. 4; to study this evolution for different *τ*, we calculate the full width at half maximum (FWHM) and an asymmetry ratio, *ξ*, of the activity band distribution as a function of *t*, depicted in Fig. 5(c and d). An example of determination of the FWHM and *ξ* is shown in Fig. 5(b) with FWHM = *x*_2_ − *x*_1_ and *ξ* = *σ*_2_/*σ*_1_. A *ξ* below 1 indicates that the majority of the rise in mobility is on the left side of the band, nearest to the moving gel edge, not the gel side of the sample. We note that the ages before the complete development of the activity band for different *τ* are neglected, as seen in Fig. 4. The activity band develops earlier for the three smaller *τ*; the width of the band increases with the macroscopic compaction of the gel. For the two larger value of *τ*, the activity band widens after the macroscopic compaction subsides with increasing *t*; this indicates a slowing down of the dynamic activity of the centre of the activity band. In terms of *ξ*, for all *τ* at all *t*, the band is asymmetric, *ξ* < 1, indicative of a wider band of relaxation on the side of the gel edge compared to the side towards the bulk. This may indicate that this side is more elastic: When stressed, the stress is distributed over a larger length for a more elastic material. We note that the noise in data of *τ*= 0.005 s at later ages can be attributed to the flattening of the band, as shown in Fig. 4.

**Fig. 6:**
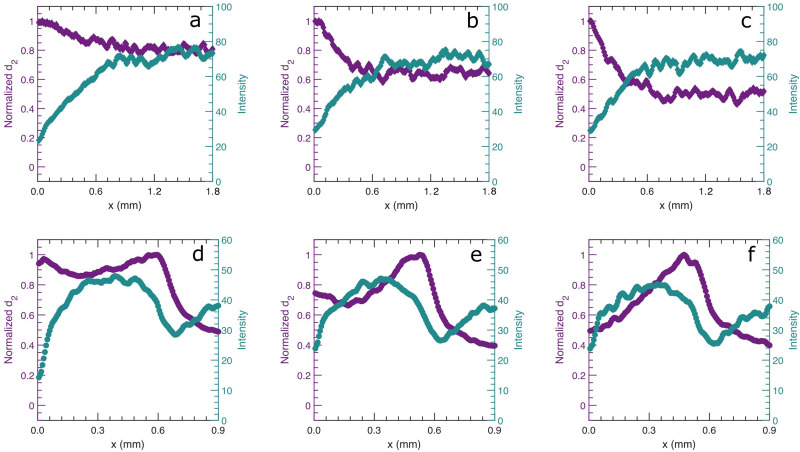
Profiles of normalized *d*_2_ and the average back-scattered light intensity as a function of distance from the moving gel edge. *x*(*t*) = 0 mm represents the moving gel edge of (**a**–**c**) the PS colloidal gel at *t* = 20, 35 and 44 min and (**d**–**f**) the PBA colloidal gel at *t* = 21, 26 and 31 min. All normalized *d*_2_ are shown for relaxation times *τ* = 0.125 seconds.

To examine the spatial variation in the density of the gel, we also monitor the average light intensity. We assume that regions of high particle density scatter more strongly so that a higher average back-scattered light intensity correlates with a higher local particle volume fraction. Both the average light intensity and the profile of normalised *d*_2_ (*τ* = 0.125 s) are shown as a function of distance from the moving gel edge in Fig. 6. In all cases, we observe that the intensity decreases towards the edge of the gel. We believe, however, that this is not caused by a decrease in local density, but rather by an asymmetry in the photon paths: while photons can reach the detector from all directions in the bulk of the sample, locations near the moving gel edge only receive photons from the right, leading to a lower photon count when *x* ≈ l* from the sample edge. Therefore, we disregard the region of lower intensity of approximately 300 μm near the edge, and consider this *x* = 0. With these reservations, there is nevertheless a significant difference between the PS and PBA gels. While in the PS gel the apparent density is more or less homogeneous, for the PBA gel, the density is reduced in the region where the dynamic activity is higher and slightly behind it. Hence, the dynamics appear to be higher in regions of lower density. The expulsion of liquid during syneresis inevitably increases the overall particle density. This process starts at the edge of the sample, as evidenced from the high dynamic activity during the first 20 minutes of syneresis as seen in Fig. 2. This increases the local density there; in the PBA gel this apparently leads to a very dense region at the front where the dynamics are slowed, probably because of the high elasticity.^33^ Further compaction must occur in the region behind this dense front, where the density is lower. By contrast in the PS gel, we find a more uniform compaction and the fastest dynamics are always localized at the edge as syneresis proceeds to later age.

### 2.3 Governing dynamics

**Fig. 7:**
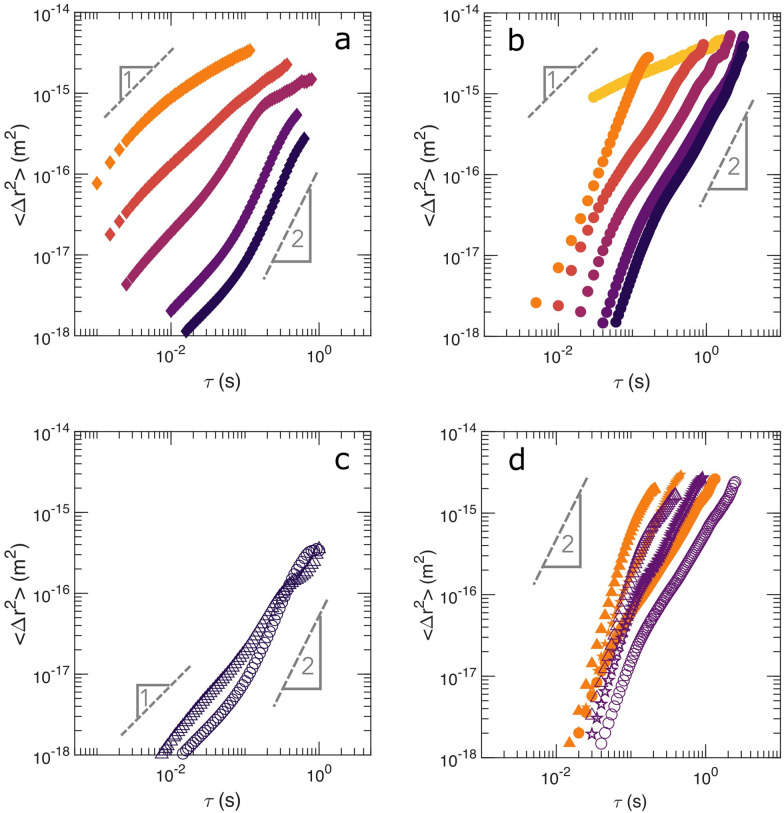
Mean squared displacement, 〈Δ*r*^2^(*τ*)〉, of (**a**) the PS gel in the bulk region during syneresis as a function of *τ*. Darkening symbol shade represents *t* = 8, 13, 20, 35 and 44 min – representative slopes of power 1 and 2 have been added as guides to the eye; (**b**) of the PBA gel in the bulk region, darkening symbol shade represents *t* = 6, 11, 16, 21, 26 and 31 min; (**c**) of the PS gel at the edge (triangle) and the bulk (circle) during syneresis at *t* = 44 min; (**d**) of the PBA gel at the activity band (triangle), valley (pentagram) and bulk (circle) during syneresis as a function of *τ*. Closed and open symbols represent *t* = 21 and 26 min, respectively.

To elucidate the nature of the observed dynamics, we extract the mean squared displacement, 〈Δ*r*^2^〉, of the particles from the LSI measurements based on the contrast function, *d*_2_. A detailed explanation is delineated in Section 4.2. The multi-speckle averaged 〈Δ*r*^2^(*τ*)〉 for selected regions at different locations in both PS and PBA gels are shown in Fig. 7. This mean squared displacement analysis inherently assumes a stationary sample, which is not the case for these syneresing networks. However, we limit this analysis to length scales of 10^−14^ m^2^ that fall within one experimental pixel, thus the displacement of the syneresing edge only weakly influences the largest displacements and *τ* while still providing a qualitative understanding of the internal dynamics. For both the PS and PBA gels, we select a rectangular area in the bulk, parallel to, but far from the moving gel edge, denoted as “bulk”. For the PBA gel, two more locations denoted as “band” and “valley” are defined, residing at the peak of the activity band and at the spatial average region between the moving gel edge and the peak of the activity band, respectively. The rectangular areas have a dimension of 0.125 × 3.0 mm for the PS gel and 0.0625 × 3 mm for the PBA gel.

For the PS gel (Fig. 7(a)), 〈Δ*r*^2^(*τ*)〉 shows diffusive behaviour during the early ages, as evidenced from the power-law slope close to 1; this diffusive behavior occurs only for very small displacement and can be interpreted as particles being thermally excited while still bound to the gel network given the magnitude of 〈Δ*r*^2^(*τ*)〉 accessibly by LSI. As the sample ages and experiences syneresis, the slope increases to around 2 for larger *τ*, indicative of ballistic motion, caused by the directional motion of the compacting gel. A transition from sub-diffusive to ballistic motion is observed in the PBA gels (Fig. 7(b)), although the initial diffusive regime is not observed here, probably because the elastic gel forms more rapidly for the PBA gel. Also, the transition to ballistic motion occurs at an earlier age than for the PS gels as syneresis begins early for these samples. From these data, we conclude that syneresis is driven by directional particle movement at the microscopic scale.

Next, we compare 〈Δ*r*^2^(*τ*)〉 in different locations in the PS and PBA gel in the regime where syneresis occurs and directional motion is observed. For the PS gel, at later ages during syneresis, 〈Δ*r*^2^(*τ*)〉 decreases gradually with increasing distance to the gel moving gel edge, indicating that the particle velocity is highest near the edge and decreases towards the bulk of the sample (Fig. 7(c)). In the PBA gel, 〈Δ*r*^2^(*τ*)〉 varies non-monotonically with the distance to the moving gel edge with 〈Δ*r*^2^(*τ*)〉 at *t* = 21 and 26 minute in the PBA gel as seen in Fig. 7(d) for valley, band and bulk regions. As seen in Fig. 5(a), the velocity of macroscopic edge displacement during syneresis in the PBA gel is approximately 2 mm/10 min ≈ 3 μm s^−1^. Considering purely ballistic motion for simplicity, 〈Δ*r*^2^(*τ*)〉 = *v*^2^ × *τ*^2^, for *τ* = 0.1 s yields 〈Δ*r*^2^(*τ*)〉 = 10^−13^ m^2^ which is close to the same order of magnitude as 〈Δ*r*^2^(*τ*)〉 in the band, shown in Fig. 7(d). As expected from the higher volume fraction and lower *d*_2_, the valley region indeed has smaller 〈Δ*r*^2^(*τ*)〉 than the band region. The smallest 〈Δ*r*^2^(*τ*)〉 is found in the bulk region, furthest from the moving gel edge in the field of view. Lastly, we note that the slope of the 〈Δ*r*^2^(*τ*)〉 appears to decrease for *τ* > 1 s for the PS gel and for the PBA gel within the activity band and bulk. This may relate to a non-uniformity in the directional motion during the macroscopic compaction of the gel, as we have observed using PIV measurements on exceedingly thin samples also undergoing syneresis^25^ This results in a more diffusive motion away from the edge, the band, the valley and then to bulk with the difference in 〈Δ*r*^2^(*τ*)〉 between different regions growing larger as *τ* increases. This difference is a direct result of the more diffusive motion of the syneresing gel from the edge approaching the bulk; as the gel compacts towards the center of the capillary, distant regions from the moving gel edge experience less directional motion during syneresis. This is possible to elucidate only with LSI due to sample turbidity.

## 3 Conclusion

We have looked at the dynamics during syneresis in colloidal gels using a non-invasive technique, LSI. The spatially resolved *d*_2_ maps allow distinguishing relative differences in the dynamics between different locations in the sample. In the PS gel, all dynamic processes have a similar spatial dependence and slow down gradually as a function of distance to the gel edge, while in the PBA gel, a pronounced activity band emerges a few hundred microns behind the gel edge. Moreover, the PBA gel exhibits spatial heterogeneity in volume fraction during syneresis; a dense region between the gel edge and the activity band is followed by a lower density zone around the activity band location. The PS gel, by contrast, shows a more uniform density. This difference between the two types of gels may be attributed to the different particle nature, *i.e.* solid or liquid.

**Fig. 8:**
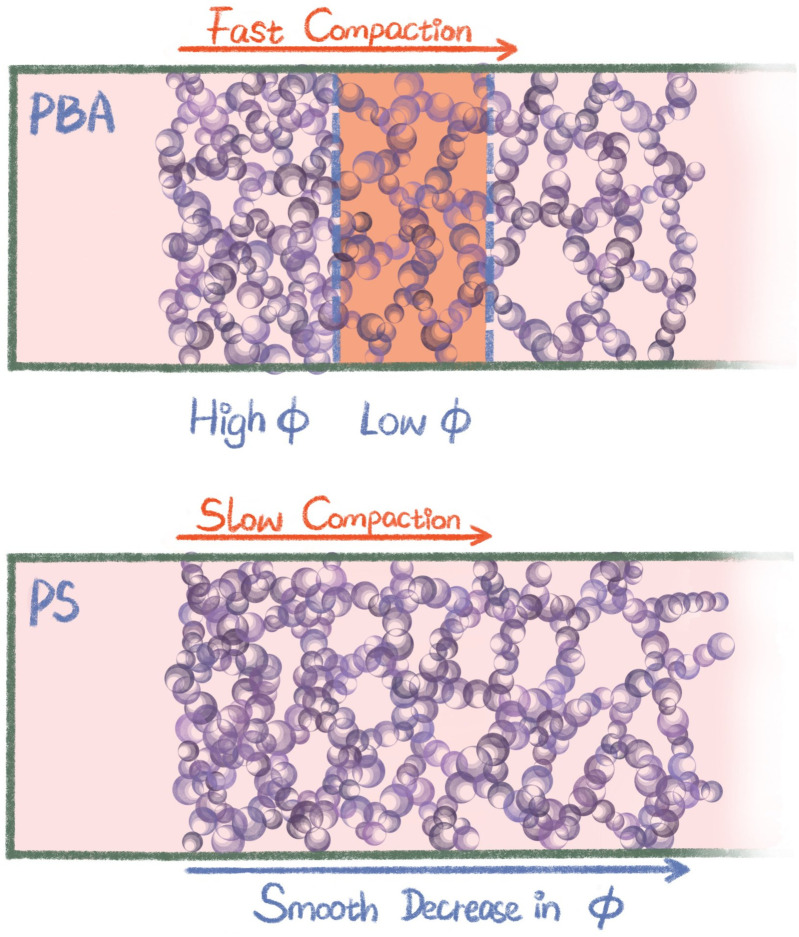
Schematic illustration of a proposed scenario for the PBA and the PS gel during syneresis.

Colloidal gels comprising of liquid particles consist of strands that can bend easily and bear stress mostly by stretching particle bonds. By contrast, solid particle gels exhibit surface asperities and a lack of surface mobility, resulting in stress being born by bending of gel strands.^25^ We propose that this difference manifests in the spatial dependence of the dynamics during syneresis, as schematically illustrated in Fig. 8. Because the elastic resistance to compaction is lower for liquid particle (PBA) gels, these gels experience fast compaction locally near the gel edge, resulting in a dense region. After a while, compaction in this region slows down as a consequence of the elastic stresses, and further syneresis proceeds in the region behind the dense zone, visible as an activity band of enhanced dynamics. By contrast, solid particle (PS) gels resist stress to a higher extent, arriving at a compaction that happens more slowly. Possibly this allows for rearrangement of the material over longer length scales, so that the compaction occurs gradually throughout the sample. These differences may explain why syneresis is most readily observed in colloidal gels composed of liquid droplets, like yoghurt and cheese, and less for solid particles like paints and coatings. However, even in solid particle based colloidal gels, syneresis may still be appreciable as shown in these PS gels.

## 4 Materials & methods

### 4.1 Sample preparation

Poly(*N*-isopropylacrylamide)(pNIPAM) surfactant is synthesized according to a published method.^27^ Polystyrene (PS) particles of 275 nm radius are synthesized using dispersion polymerisation of styrene (Sigma) at 80 °C for 2 hours using AIBN (Sigma) as a radical initiator.^34^ The dispersion is filtered and washed by centrifugation (3×) into 0.1 wt% pNIPAM surfactant and 100 mM NaCl solution to a final *ϕ* = 0.2. The recipe for the synthesis of polybutylacrylate (PBA) droplets by UV initiated emulsion polymerization is as follows: a disperse phase, comprising of butyl acrylate with 0.3 wt% 2,2-dimethoxy-2-phenylacetophenone as initiator, and the continuous phase, comprising of 3 mM NaCl and 1 wt% pNIPAM, are mixed with a ratio 1/4 (v/v%). The mixture is vortexed and then bath-sonicated at 10 °C for 25 min. After bubbling with *N*_2_ for 10 min in a sealed vial, the mixture is exposed to UV light for 10 min while gently mixing at 10 °C. The mixture is left overnight for complete polymerization at room temperature. PBA has a low glass transition temperature (*T*_*g*_) and thus exists as liquid at room temperature. The particles have an average radius, 〈*r*〉 = 275 nm, as determined by dynamic light scattering. PBA droplets are filtrated and dialyzed against 0.1 wt% pNIPAM surfactant and 100 mM NaCl solution to a final *ϕ* = 0.2 approximately.

A polyelectrolyte multilayer, PEM, coating is prepared according to the following procedure^35^: solutions of 1 wt% cationic polymer, polydiallyldimethylammonium chloride (*M*_w_ = 4 × 10^5^ − 5 × 10^5^ g mol^−1^, Sigma), in 1 M NaCl and 1 wt% anionic polymer, sodium polystyrene sulfonate (*M*_w_ ∼ 2 × 10^5^ g mol^−1^, Sigma) in 1 M NaCl are prepared. The solutions are filtered with 0.45 μm hydrophilic filter. To begin the process, the glass container is cleaned using a plasma cleaner. This makes the glass anionic, negatively charged. Cationic polymer solution is first added to the container and then rinsed away with sufficient amount of deionized water by filling the container completely many times and then leaving it empty after 5 minutes. Cationic and anionic layers are added alternatively for 3 layers each with thorough rinsing in between. This PEM coating prevents adhesion of the colloidal gel to the wall. Further description of the sample preparation and dynamics can be found in a previous publication.^25^

Dispersions of PS particles, *ϕ* = 0.01, and PBA droplets, *ϕ* = 0.02, are injected into PEM coated rectangular cross-section capillaries (Rectangle Boro Tubing, 9.0 × 40.0 mm × 0.9 mm) (*x*, *y*, *z*). At both ends of the capillary a hydrophobic paste (Krytox GPL205) is applied to prevent adhesion to the epoxy gel (Devcon) for capillary closure. This closer pinned the dispersion, resulting in syneresis occurring perpendicular to the length of the capillary as shown in Fig. 1. This resulted in the observed 1-dimensional syneresis, *i.e.* from the capillary wall to the midpoint of the capillary; without this pinning, syneresis would be 2-dimensional and challenging to interpret due to sample drift within the capillary. During the LSI measurement, samples are situated in a thermostatic chamber at 32 °C.

Experiments of both samples were conducted *N* > 5 times; while the discussion above concerns only a single experimental instance, the LSI results of the repetitions were highly reproducible in regards to the appearance, distance from the moving edge, and shape of the activity band for the PBA sample. In no repetition was an activity band seen in the PS gel. Some variation between repetitions was seen in the magnitude of syneresis, *i.e.* final distance as shown in Fig. 5a, due to presumed inconsistencies with heating and pinning at the capillary entrances; the variation was <0.5 mm between repetitions.

### 4.2 Laser speckle imaging and analysis

The set-up of LSI is illustrated schematically in Fig. 1 with further technical details of the set-up are discussed elsewhere.^16^ Briefly, coherent light (*λ* = 532 nm, Cobolt Samba, 1 W) is expanded to a diameter of 1 cm by a Galilean beam expander and illuminates the sample being directed downward onto the sample *via* two mirrors, at a small angle with respect to the detection path to avoid intensity enhancement by coherent backscattering. The backscattered light is reflected by a mirror onto a linear polariser perpendicular to the polarisation of the incident laser beam and collected by a Qioptiq zoom lens, 1.8× and the depth-of-focus is 0.1 mm, and focused through an iris diaphragm with the speckle size tuned by the diaphragm to be slightly larger than the pixel size, typically 2–3 pixels. The back-scattered light is then captured by one of two cameras discussed below. The samples are situated on a platform which is connected to a computer-controlled balance (Sartorius, model WZA224-NC), logging the sample mass with 0.1 mg resolution at 1 Hz frequency to confirm that sample evaporation does not occur. A climate chamber is applied to the set-up, which controls the ambient temperature of the sample, as well as minimizes air convection and stray light. Climate control also minimizes sample drift which was observed to be less than 1 pixel by monitoring the position of the capillary wall within the image field of view.

The laser power, exposure time and frame rate are important for capturing the right information. To prevent blurring of the speckles and to capture fast changing speckles, the exposure time is set as short as possible where it could still cover the full dynamic range of the camera. This minimizes over- and under-exposure hence data loss. Two different cameras are used: (i) Dalsa Genie camera (Stemmer Imaging) for the PBA colloidal gel at 200 fps and 100 fps, respectively. (ii) HiSpec 1 camera (Fastec Imaging) for the PS colloidal gel at 2000 fps. The field of view is 640 × 480 pixels (4.0 × 3.0 mm) with the Dalsa Genie camera and 640 × 240 (8.0 × 3.0 mm) with the HiSpec 1 camera.

The contrast function, *d*_2_ (eqn (1)) is calculated to resolve dynamics during syneresis, which vary significantly in both time and space. Symmetric normalization, *i.e.* the denominator with the product of mean intensities at times *t* and *t* + *τ* is used instead of the square of the mean intensity, which diminishes intensity drift-induced artefacts.^16^ Values of *d*_2_ are averaged over 0.45 s to improve statistical analysis.

Note that fast macroscopic movement of the syneresing colloidal gels, and the thin edge, is prone to induce too low intensity in the speckle images at the moving gel edge, resulting in high *d*_2_ noise artefacts. All calculations and analysis in this work are carried out on speckle images where a small integer value, Δ*I* = 5, is added to all pixels based on *d*_2_ noise check and elaborated in the ESI.†

To achieve calculation of 〈Δ*r*^2^(*τ*)〉, *d*_2_ is first converted into the electric field correlation function, *g*_1_, according to 
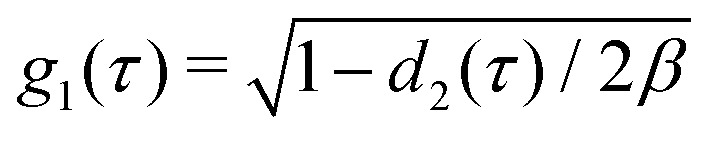
. This is based on the Siegert relation that, 
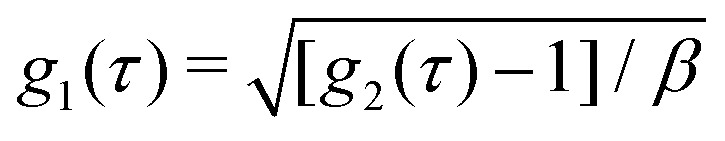
 and the fact that *d*_2_(*τ*) ≈ 2[*g*_2_(*τ* = 0) − *g*_2_(*τ*)].^30^*β* is the spatial coherence factor, accounting for the number of speckles detected. In the ideal case, when fluctuations of only a single speckle are detected in each pixel, *β* equals 1.^31^ Due to the limitations in camera-based detection, *β* < 1. Here *β* is determined as the limit of *τ* → 0, *β* = *g*_2_(*τ*) − 1; typical values are between *β* = 0.6–0.7. The mean squared displacement, 〈Δ*r*^2^(*t*,*τ*)〉, can then be extracted based on:2
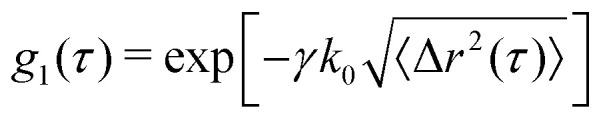
where *k*_0_ = 2π*n*/*λ* is the wave vector, with *n*, being the refractive index of water, *λ*, being the wavelength of the impinging coherent light and *γ* is a numerical prefactor that is obtained from literature to be 1.5.^15,16,36^

The photon transport mean free path, l*, or the distance within a sample a photon must travel to eliminate any directional memory, for both samples was measured using a relative transmitted light intensity to a sample of known l* and thickness, *L*; according to,3
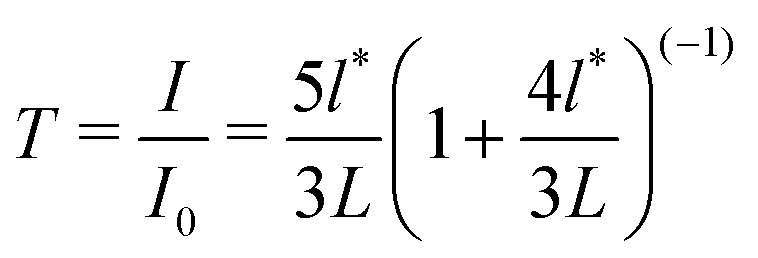


The initial values for *ϕ* = 0.01 PS dispersion l* = 132 μm and *ϕ* = 0.02 PBA dispersion l* = 115 μm are significantly smaller than the sample capillary thickness, 0.9 mm with *L* ≈ 10l*. The value of l* decreases during syneresis as *ϕ* locally increases. These values, combined with the crossed linear polariser, ensure that photons collected by the camera are multiply scattered and diffusive and the application of eqn (2) is appropriate.

## Conflicts of interest

There are no conflicts to declare.

## Supplementary Material

SM-019-D3SM00448A-s001
