# Supplementary material for: Spatially heterogenous dynamics in colloidal gels during syneresis
Source: Soft Matter. 2023 Jun 29;19(28):5336–44. doi: 10.1039/d3sm00448a (PMC10355138; doi:10.1039/d3sm00448a)
Supplement: SM-019-D3SM00448A-s001 [file SM-019-D3SM00448A-s001.pdf]

Electronic Supplementary Material (ESI) for Soft Matter.
This journal is © The Royal Society of Chemistry 2023

Journal Name

ARTICLE TYPE

Cite this: DOI: 10.1039/xxxxxxxxxx

Electronic Supplementary Information to: Unravelling Spatially Heterogenous Dynamics in Colloidal Gels during Syneresis

Qimeng Wu, Jesse Buijs, Sanne de Groot, Hanne van der Kooi, Jasper van der Gucht and Thomas E. Kodger*

1 Syneresis Movies of Colloidal Gels

Movie 1 = "d_2 maps of PS gel during syneresis, τ=0.125 s, colormap inferno. Left end of d_2 maps represents the capillary wall. 2fps"
Movie 2 = "d_2 maps of PBA gel during syneresis, τ=0.125 s, colormap inferno. Left end of d_2 maps represents the capillary wall. 2fps"

2 Noise Artefacts Check at the Gel Edge

Fast macroscopic movement of the syneresing colloidal gels is prone to induce too low intensity in the speckle images at the moving gel edge, resulting in high d_2 noise artefacts. To check for this possible effect, a series of images with ΔI = 1, 2, 5 and 8 added to the speckle images for the PBA gel are shown in Fig. S1. Intensity fluctuations in properly illuminated areas are conserved but the fluctuations in poorly illuminated areas are less overestimated.

With increasing ΔI, the high d_2 stripe, indicating high mobility, at the sample moving edge vanishes and a stripe with low mobility, low d_2 developed indicating that the high d_2 is indeed related to noise artifacts. This may be related to incomplete randomization of photons in this area due to the fast macroscopic movement and the resulting thin edge. Therefore, all calculations and analysis in this work are carried out on speckle images with ΔI =5.

[a] Physical Chemistry and Soft Matter, Wageningen University and Research, Stippeneng 4, 6708 WE, Wageningen, The Netherlands . E-mail: thomas.kodger@wur.nl

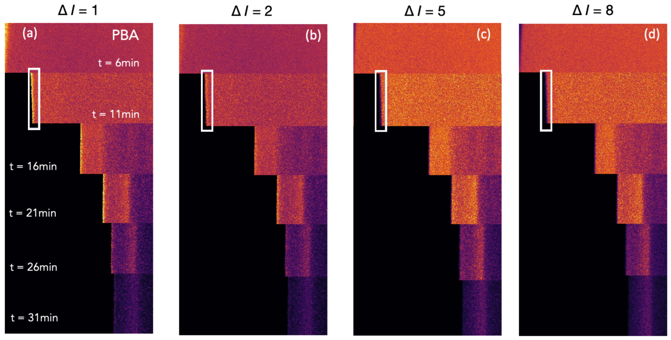

Fig. S1 Time-lapse d_2 maps of PBA gel, τ=0.125 s, calculated from ΔI = 1, 2, 5 and 8 to the speckle images, colormap inferno. Left end of d_2 maps represents the capillary wall. Region of interest with low intensity at the moving edge of the gels is marked with white rectangles for t = 11 min.
